# Hypothalamic pituitary thyroid axis and exposure to interpersonal violence in childhood among women with borderline personality disorder

**DOI:** 10.3402/ejpt.v5.23911

**Published:** 2014-05-16

**Authors:** Cave Sinai, Tatja Hirvikoski, Anna-Lena Nordström, Peter Nordström, Åsa Nilsonne, Alexander Wilczek, Marie Åsberg, Jussi Jokinen

**Affiliations:** 1 Department of Clinical Neuroscience/Psychiatry, Karolinska Institutet, Karolinska University Hospital, Solna, Sweden; 2 Department of Children's and Women's Health, Center for Neurodevelopmental Disorders at Karolinska Institutet (KIND), Stockholm, Sweden; 3 Department of Clinical Sciences, Karolinska Institutet, Danderyd Hospital, Stockholm, Sweden

**Keywords:** HPT axis, thyroid hormones, borderline personality disorder, interpersonal violence, childhood adverse events, childhood maltreatment, trauma, stress

## Abstract

**Background:**

A relationship between exposure to sexual violence and thyroid hormone alterations has been observed among women with posttraumatic stress disorder (PTSD). Women with borderline personality disorder (BPD) report a high estimate of childhood trauma.

**Objective:**

The aim of the present study was to assess relationships between thyroid hormone measures and exposure to violence in childhood in women with BPD.

**Method:**

A total of 92 clinically euthyroid women with BPD (53% with comorbid PTSD) diagnosis and at least two prior suicide attempts were assessed with the Karolinska Interpersonal Violence Scales (KIVS). The KIVS contains four subscales with concrete examples of exposure to violence and expressed violent behavior in childhood (aged 6–14 years) and during adult life (15 years or older). Baseline thyroid function was evaluated by measuring plasma free and bound triiodothyronine (FT3 and T3), thyroxine (FT4 and T4), and thyroid-stimulating hormone (TSH) with immunoassays. The FT3/FT4 ratio was used to estimate peripheral deiodination. Plasma cortisol was also assessed.

**Results:**

Sixty-seven percent of patients reported medium high or high level of exposure to interpersonal violence as a child. The FT3/FT4 ratio showed a significant negative correlation with exposure to violence as a child. Patients with PTSD had significantly higher plasma cortisol levels. An ad hoc analysis revealed that the correlation between KIVS exposure to interpersonal violence as a child and FT3/FT4 ratio was significant only in patients with comorbid PTSD. Altered thyroid activity, especially FT3/FT4, levels was associated with exposure to violence in childhood in women with BPD.

**Conclusion:**

Severe childhood trauma-related stress may promote lasting altered thyroid levels and/or contribute to the development of psychopathology associated with BPD traits or PTSD.

Borderline personality disorder (BPD) is a burdening condition characterized by affective instability, identity disturbance, instability of interpersonal relationships, self-image and affect, as well as destructive impulsive behavior, such as substance abuse and high suicide risk (Yen et al., 2004). The estimated prevalence in population-based studies is 0.5–5.9% (Grant et al., 2008; Samuels et al., 2002). More than 90% of individuals diagnosed with BPD report exposure to traumatic experiences (Yen et al., 2002; Zanarini et al., 1997) and the rate of comorbid posttraumatic stress disorder (PTSD) varies between 25 and 58% (Golier et al., 2003; Zanarini, Frankenburg, Hennen, Reich, & Silk, 2004). In particular, the cluster of features comprising impulsivity, mood reactivity, self-injury, suicidality, and dissociation has been proposed to illustrate the susceptibility of BPD patients to maladaptive stress responses (Zimmerman & Choi-Kain, 2009). Adult personality psychopathology (Brodsky, Malone, Ellis, Dulit, & Mann, 1997) and suicide attempts as well as completed suicides are associated with childhood trauma in the form of severe neglect (Glaser, 2000) and sexual or physical abuse (Dinwiddie et al., 2000; Jokinen et al., 2010; Sunnqvist, Westrin, & Träskman-Bendz, 2008; Ystgaard, Hestetun, Loeb & Mehlum, 2004), probably mediated through dysregulated neurobiological stress systems (De Bellis, Spratt, & Hooper, 2011), aggressive traits (Sarchiapone et al., 2009), and family dynamics (Brodsky et al., 2008). It has been suggested that childhood maltreatment may mold the phenotypic characteristics of psychopathology, yielding a variety of ecophenotypes (Teicher & Samson, 2013).

The hypothalamus–pituitary–thyroid (HPT) axis has been less studied than the hypothalamic–pituitary–adrenal (HPA) axis in stress research (Olff, Guzelcan, De Vries, Assies, & Gersons, 2006). The thyroid hormones triiodothyronine (T3) and thyroxine (T4) are secreted from the thyroid gland in response to thyroid-stimulating hormone (TSH), released from the anterior pituitary gland. TSH is regulated by thyrotropin-releasing hormone (TRH) released from the hypothalamus. Increased T4 levels are involved in the stress arousal indicating involvement of the HPT axis in stress regulation and disorders in the stress system.

Few studies have investigated the HPT axis function in relation to reported traumatic experiences among individuals with personality disorders. Most of the studies have focused on two clinical groups: women with a history of childhood sexual abuse (with or without PTSD) and individuals suffering from combat related PTSD (Wang, 2006). Earlier studies have reported elevated levels of free and total triiodothyronine (FT3 and T3) in individuals with PTSD as compared to a control group (Karlovic, Marusic, & Martinac 2004; Kozaric-Kovacic, Karlovic, & Kocijan-Hercigonja, 2002; Mason et al., 1994; Wang & Mason, 1999).

Among individuals with BPD, dysregulated HPA Axis functioning has been observed with suppression in feedback inhibition in comorbid major depression, and enhanced feedback inhibition with concurrent diagnosis of PTSD and self-aggressive behaviors (Zimmerman & Choi-Kain, 2009). Most of the studies of plasma cortisol levels in individuals with PTSD have shown lower cortisol levels, and lower daily cortisol output may be associated with PTSD in particular (Morris, Compas, & Garber, 2012). However, several studies included in the meta-analysis of Meewisse, Reitsma, De Vries, Gersons, and Olff (2007) have reported higher cortisol levels in PTSD patients, indicating that exposure to trauma is related to a dysregulation of the HPA axis.

## Aim of the present study

The aim of the present study was to assess the relationship between exposure to interpersonal violence as a child and thyroid hormones in female patients with BPD. We measured free and bound fractions of T3 and T4 and TSH, as well as plasma cortisol, in female patients with BPD and a history of suicide attempts. We hypothesized that exposure to interpersonal violence as a child would be associated with altered adult thyroid hormone levels. Since most of the literature relating HPT dysregulation to trauma has involved patients with PTSD, we further analyzed the patients with and without comorbid PTSD diagnosis separately.

## Method

### Study setting

Female patients with a diagnosis of BPD were recruited between 1999 and 2004 for a psychotherapy treatment outcome study: “Stockholm county council and Karolinska Institute Psychotherapy project for suicide-prone women” (SKIP project). The SKIP project is a randomized controlled trial, comparing the efficacies of two forms of psychotherapy, and general psychiatric care (treatment as usual). The Regional Ethical Review Board in Stockholm approved the study protocol (Dnr. 95-283) and the participants gave their written informed consent to the study.

Inclusion criteria were a history of at least two suicide attempts (defined as a self-destructive act with some degree of intent to die), borderline diagnosis according to DSM–IV, a fair capacity to communicate verbally and in writing in the Swedish language, and aged between 18 and 50 years. Exclusion criteria were schizophrenia spectrum psychosis, melancholia, mental retardation, drug abuse and severe anorexia.

### Patients

A total of 162 women with BPD were invited to take part in the SKIP project. Of these individuals, 14 declined to join the study, 41 were excluded due to not fulfilling inclusion criteria or to fulfilling exclusion criteria and one completed suicide before joining the study. Thus, out of 162 women, 106 (65%) took part in the SKIP study. We obtained laboratory data for 97 of 106 individuals; 92 patients were euthyroid (TSH reference range: 0.4–3.5 mE/l, Karolinska University Hospital) and thus included in the statistical analyses described below.

The mean age of the patients was 29.5 years (SD=7.6; range 19–50). The participants were interviewed by a trained psychiatrist, using the SCID I research version interview to establish the DSM–IV diagnoses (First, Spitzer, Gibbon, & Williams, 1997). Trained clinical psychologists established Axis II diagnoses by DIP–I interviews (Ottosson et al., 1995). All self-rating scales were completed under the supervision of a research nurse. 90 (98%) of the participants had at least one current Axis I psychiatric diagnosis. Among the Axis I diagnoses, 78 (85%) of the patients met the criteria for mood disorders (unipolar major depressive disorder, single episode or recurrent, bipolar disorder, depressed or dysthymic disorder), 76 (83%) for anxiety disorders. Forty nine out of 92 (53%) patients met the criteria of PTSD. 24 (26%) had a comorbid eating disorder; of whom 16 (17%) with bulimia and eight (9%) with anorexia nervosa. Eight women (9%) had a diagnosis of alcohol abuse. Fifty women had an additional personality disorder (PD); avoidant PD (*n*=24), paranoid PD (*n*=15), obsessive–compulsive PD (*n*=12), histrionic PD (*n*=10), dependent PD (*n*=9), narcissistic PD (*n*=4). 22 (24%) women had three or more personality disorders. The criteria for conduct disorder were met in seven of the women. Medication records were obtained for 68 (74%) of the patients, of whom seven patients were medication-free. Three patients were treated with lithium. The most frequent medications were venlafaxin (*n*=12), fluoxetine (*n*=11), sertraline (*n*=9) and citalopram (*n*=4). Two patients had a combination of two antidepressants.

### Neuroendocrine testing

Baseline thyroid function was evaluated by measuring plasma free and bound T3, T4 and TSH levels. Venous blood was drawn and immediately frozen in aliquots at −70°C or below until analyzed. The samples were thawed and analyzed by immunoassays (Unicel DxI 800 Beckman Coulter, for FT4, FT3 and TSH and AutoDelfia, for T4 and T3) in the year 2010. No prior thawing of the frozen plasma samples had been performed. The Karolinska Laboratory at Karolinska University Hospital performed all analyses according to accredited routines. The intra-assay coefficient of variation for TSH was 3.85–5.56%, for FT4 2.74–4.4%, for FT3 5.1–6.6%, for T4 2.7–3.6% and for T3 2.9–3.1%. The inter-assay coefficient of variation for TSH was 3.02–3.68%, for FT4 3.34–8.08%, for FT3 1.3–8%, for T4 1.4–2.2% and for T3 1.2–2.1%. Analytical interferences in thyroid hormone testing are estimated to occur in less than 0.1%, at the Karolinska Clinical Laboratory. The FT3/FT4 ratio was used to estimate peripheral deiodination. Preanalytical variation was minimized by performing the venipuncture in a standardized manner for all participants, of which the majority was sampled at noon.

### Assessments

The Karolinska Interpersonal Violence Scale (KIVS) (Jokinen et al., 2010) contains four subscales with direct questions with concrete examples of exposure to violence and expressed violent behavior in childhood (aged 6–14 years) and during adult life (15 years or older). The ratings are filled in during a structured interview to elicit a comprehensive lifetime trauma and victimization history and history of lifetime expressed violent behavior. Interviews and ratings (0–5 for each subscale, total 20) were performed and assessed by trained psychiatrists. The inter-rater reliability of the KIVS subscales was high (*r*>0.9). The KIVS scale has been validated against several other rating scales measuring aggression and acts of violence (Jokinen et al., 2010). The KIVS scores were grouped into three levels of exposure to interpersonal violence as a child: score 0 and 1 representing “no exposure or mild level of exposure,” score 2 and 3 representing “medium high level of exposure,” and score 4 and 5 representing “high level of exposure” (see Table 1).

**Table 1:** The KIVS scores grouped into three levels of exposure

The KIVS score	The KIVS statements	Classification in current study
0	No violence.	
1	Occasional slaps. Fights in school, of no great significance.	Low
2	Bullied occasionally for short period(s). Occasionally exposed to corporal punishment.	Medium high
3	Often bullied. Frequently exposed to corporal punishment. Beaten by drunken parent.	
4	Bullied throughout childhood. Battered/beaten up by schoolmates. Regularly beaten by parent or other adult. Beaten with objects. Sexually abused.	High
5	Repeated exposure to violence at home or in school that resulted at least once in serious bodily harm. Repeated sexual abuse, or sexual abuse that resulted in bodily harm.	

### Statistical analysis

One female was identified as both a univariate and a multivariate outlier. The exclusion of this individual did not affect the results and she was therefore included in all analyses. Correlation analyses were used to determine associations between the clinical ratings and biological variables. Initially, tests of nonparametric or parametric correlations were performed using Spearman's rho or Pearson's r. Some variables were analyzed by both non-parametric as well as parametric statistical methods due to the non-normality of sample distributions; however, the choice of statistical method did not change the results. Therefore, for the sake of brevity we only report parametric statistics. Group differences were computed with one-way ANOVA or with Wilcoxon test in continuous variables. For categorical variables, the group comparisons were calculated using the Chi-square test. Effect sizes for significant Chi-square tests were expressed as Kendall's tau-c and interpreted as weak association (0.10–0.20), moderate association (0.20–0.40), relatively strong association (0.40–0.60), strong association (0.60–0.80), or very strong association (0.80–1.00). If the expected number was <5 in any of the cells, the Chi-square test was not performed. Based on the results of bivariate analyses, the association between FT3/FT4 ratio and exposure to interpersonal violence as a child was computed with multiple regression analysis adjusted for age, PTSD, plasma cortisol and sample storage time. The residual scatterplots were examined to check the assumptions of normality, linearity and homoscedasticity between the predicted dependent variable scores and errors of prediction, and the assumptions were deemed to be satisfied. Furthermore, the Durbin-Watson test statistic expressed no correlation in adjacent residuals and the variance inflation factor (VIF) and tolerance statistic indicated no problem with multicollinearity. In order to preserve most of the sample size and thereby statistical power, missing data was handled by pairwise deletion in the statistical analyses. The alpha level was set on <0.05 while *p*-values 0.05<*p*<0.10 were regarded as statistical trends. The statistical analysis was performed using the SPSS statistical software package (IBM, SPSS™, version 22).

## Results

### Clinical assessments

**Fig. 1:**
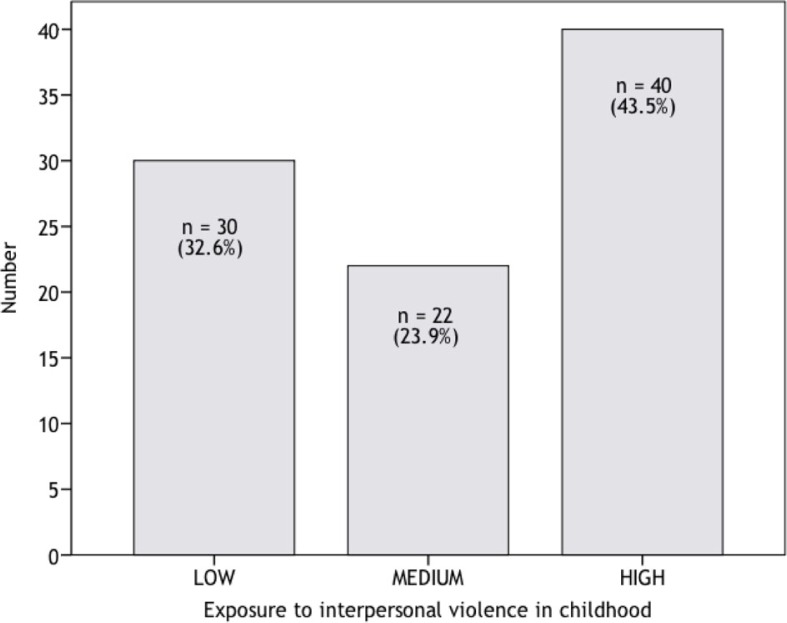
Number of individuals with exposure to interpersonal violence in childhood, among women with borderline personality disorder.

KIVS ratings were available from 92 euthyroid participants. The mean exposure to interpersonal violence as a child was 2.6 KIVS scale points (SD=1.9, median=3, range 0–5). Numbers of KIVS ratings are depicted in Fig. 1. There were no significant differences between the three exposure groups concerning age, any use of medication or frequency of Axis I diagnoses. PTSD diagnosis was significantly more frequent in the high exposure group as compared to the low (*p*<0.05) and the medium high groups (*p*<0.05) (see Table 2).

**Table 2:** Clinical characteristics of the three KIVS groups

KIVS group	Low (*n*=30)	Medium high (*n*=22)	High (*n*=40)	Statistics	*p*
Mean age (SD)	28 (±6.75)	30 (±7.80)	30 (±8.1)	*F* (2,89)=0.67	0.52
Axis I diagnose	29 (97%)	21 (95%)	40 (100%)	*χ* ^2^=0.06	0.81
Additional Axis II diagnose (1 or more)	12 (40%)	15 (68%)	23 (58%)	*χ* ^2^=4.04	0.13
PTSD	10/30 (33%)	8/22 (36%)	31/40 (78%)	*χ* ^2^=16.75, *τ*=0.43	**0.0002**
				*χ* ^2^=13.78, *τ*=0.43	**0.0002** (L vs. H)
				*χ* ^2^=10.29, *τ*=0.38	**0.001** (M vs. H)
				*χ* ^2^=0.05, *τ*=0.03	0.82 (L vs. M)
Medication, any psychoactive medication	16 (53%)	14 (64%)	28 (70%)	*χ* ^2^=2.04	0.36
Antidepressants	14 (46%)	11 (50%)	20 (50%)	*χ* ^2^=0.09	0.96
Lithium	1	2	0	⊗	
No medication	2	2	3	⊗	
No registered records of medication	10 (33%)	5 (23%)	9 (23%)	*χ* ^2^=1.2	0.55

All estimated shown in bold are significant at *p<*0.05. KIVS=Karolinska Interpersonal Violence Scale, L=low: KIVS rating 0–1, M=medium high: KIVS rating 2–3, H=high: KIVS rating 4–5. ⊗=statistical analysis was not performed. No registered records of medication=data missing due to changes in research protocol. *τ* = Kendall's tau-c.

### Neuroendocrine measures and exposure to interpersonal violence as a child and the regression model for the FT3/FT4 ratio

**Fig. 2:**
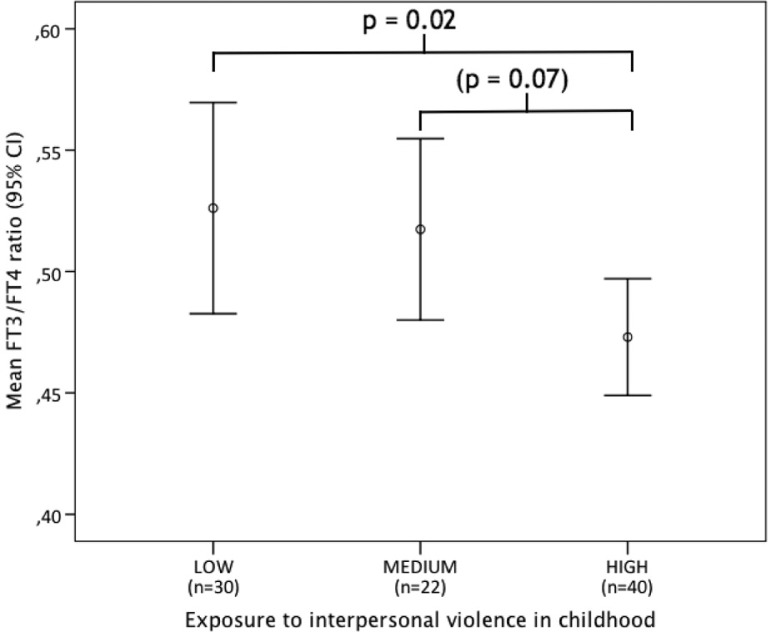
FT3/FT4 ratio in female BPD patients with low, medium high, and high levels of exposure to interpersonal violence as a child.

**Fig. 3:**
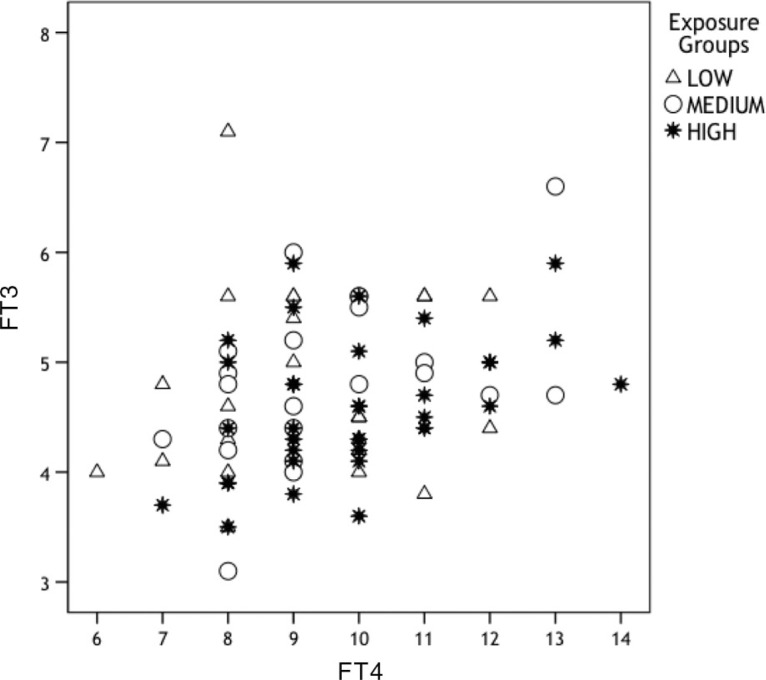
Correlation between FT3 (pmol/L) and FT4 (pmol/L) in the three groups of KIVS exposure to interpersonal violence as a child.

Among participants, a significant difference between the exposure groups was found (F (2, 89)=3.3, *p*=0.04). Post hoc analysis with the LSD test showed that the high exposure group had significantly lower FT3/FT4 ratio (mean=0.47, SD=0.08) as compared with the low exposure group (mean=0.53, SD=0.12) (*p*=0.02) (see Fig. 2). Figure 3 shows the correlation between FT3 and FT4 in the three groups of KIVS exposure to interpersonal violence as a child.

The correlation between KIVS exposure to interpersonal violence as a child and the FT3/FT4 was significant (Pearson's *r*=−0.25, *p*=0.02), and was therefore further analyzed in the multiple regression analysis. The correlation matrix and results of the multiple regression analysis are presented in Table 3. A multiple regression of FT3/FT4 as dependent variable and KIVS exposure to interpersonal violence as a child, age, PTSD diagnosis, serum cortisol and sample storage time, as independent variables, was performed. The overall model was significant (*F* (5, 85)=5.1, *p*=0.0004) with *R*=0.48, *R* ^2^=0.23 and adjusted *R* ^2^=0.18, which implies that the model accounted for 18% of the variance in FT3/FT4 ratio.

**Table 3:** Correlation matrix and results of the multiple regression analysis for KIVS exposure to interpersonal violence in childhood and FT3/FT4 in female patients with borderline personality disorder

	FT3/FT4	KIVS exposure	Age	PTSD	Cortisol	*B*	*SE B*	*β*
KIVS exposure	−0.25*					−0.019	0.005	−0.38*
Age	−0.06	0.1				0.001	0.001	0.1
PTSD	0.14	0.36*	−0.02			0.02	0.02	0.26*
Cortisol	0.21*	0.08	−0.26*	0.29*		0.000	0.000	0.16
Sample storage time	0.24*	0.05	−0.26*	−0.09	0.11	0.000	0.000	0.29*

All tests are two-tailed. KIVS Exposure = Karolinska Interpersonal Violence Scale; exposure to interpersonal violence in childhood.

* *p*<0.05.

Three independent variables were statistically significant predictors of the FT3/FT4 ratio: KIVS exposure to violence as a child (*p*=0.0004), diagnosis of PTSD (*p*=0.018) and sample storage time (*p*=0.005). The standardized value of B (for KIVS childhood interpersonal exposure to violence)=−0.38 indicates a negative relationship to the FT3/FT4 ratio; thus, higher scores on KIVS exposure to interpersonal violence were associated with a lower FT3/FT4 ratio. The Durbin-Watson statistic was 2.1, thus the assumption of independent errors was met. In the final regression model, dropping the non-significant predictors age and cortisol, the overall model was significant (*F* (3, 87)=7.4, *p*=0.0002) and still accounts for 17.4% of the variance of the FT3/FT4 ratio.

### Diagnosis of PTSD in relation to hormone levels

**Fig. 4:**
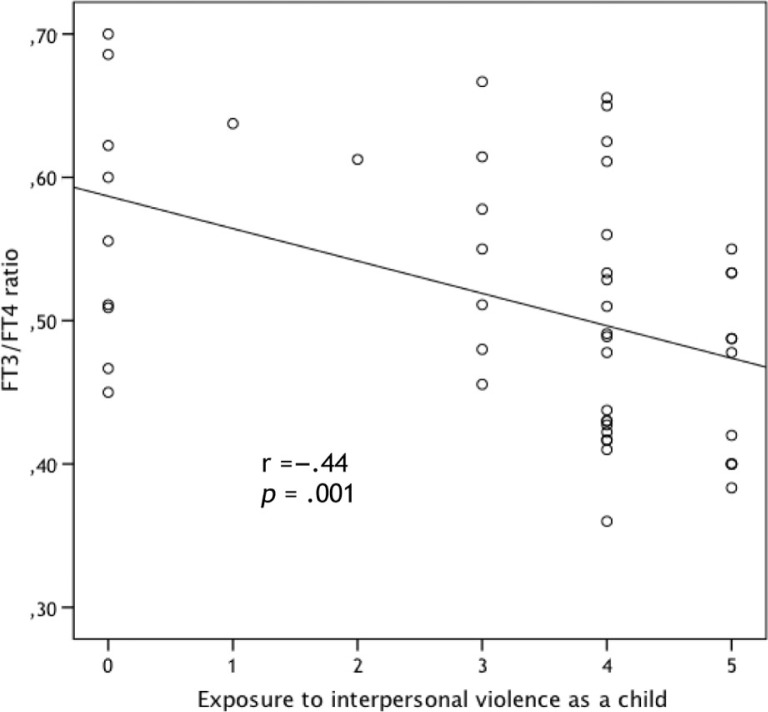
Correlation between KIVS exposure to interpersonal violence as a child and FT3/FT4 ratio, in patients with comorbid PTSD.

There were no significant differences in thyroid hormone levels in female BPD patients with and without comorbid PTSD diagnosis (Table 4). Patients with PTSD had significantly higher plasma cortisol levels (*Z*=−2.4, *p*=0.014). The correlation between KIVS exposure to interpersonal violence as a child and FT3/FT4 ratio was significant only in patients with comorbid PTSD (Pearson's *r*=−0.44, *p*=0.001) (see Fig. 4). Furthermore, in patients with comorbid PTSD, exposure to interpersonal violence as a child also showed a significant negative correlation with FT3 (Pearson's *r*=−0.41, *p*=0.004).

**Table 4:** Group comparisons (Wilcoxon and *t*-test) of hormone levels in female borderline patients with and without comorbid PTSD diagnosis

	PTSD	Non-PTSD	
Variables	Mean (SD) Median (Range) *n*	Mean (SD) Median (Range) *n*	Statistic
TSH	1.4 (0.7) 1.2 (0.5–3.0) *n*=49	1.3 (0.7) 1.1 (0.4–3.5) *n*=43	*Z=*−0.9 (*p=*0.35)
T3	1.9 (0.3) 1.8 (1.4–2.5) *n*=43	1.9 (0.4) 1.9 (1.2–2.5) *n*=40	*Z=*−0.1 (*p=*0.96)
T4	104 (20) 105 (65–140) *n*=45	102 (16) 103 (70–135) *n*=39	*t*=0.5 (*p=*0.64)
FT3	4.7 (0.6) 4.8 (3.5–6) *n*=49	4.6 (0.8) 4.5 (3.1–7.1) *n*=43	*Z=*−1.2 (*p=*0.23)
FT4	9.4 (1.4) 9 (7–13) *n*=49	9.7 (1.7) 10 (6–14) *n*=43	*Z=*−1.0 (*p*=0.31)
T3/T4	0.02 (0.003) 0.02 (0.01–0.02) *n*=42	0.02 (0.003) 0.02 (0.01–0.02) *n*=39	*Z=*−0.2 (*p=*0.84)
FT3/FT4	0.5 (0.1) 0.5 (0.4–0.7) *n*=49	0.49 (0.1) 0.46 (0.34–0.89) *n*=43	*Z=*−1.6 (*p=*0.12)
Cortisol	458 (224) 429 (140–1,070) *n*=49	346 (127) 340 (168–600) *n*=43	*Z=*−2.4 (*p=*0.014)

TSH (mE/L), T3 (nmol/L), T3 (pmol/L), T4 (nmol/L), FT3 (pmol/L), FT4 (pmol/L), cortisol (nmol/L).

## Discussion

In the present study, we measured thyroid hormones and cortisol levels in female patients with BPD and a history of suicide attempts. We measured the exposure to interpersonal violence with the Karolinska Interpersonal Violence Scale. Two thirds of the patients reported medium high or high levels of exposure to interpersonal violence as a child, which is in line with studies reporting frequent trauma exposures (up to 70%) in individuals diagnosed with BPD (Paris, Zweig-Frank, & Guzder, 1994; Yen et al., 2002). More than half of the patients fulfilled the DSM criteria for comorbid PTSD. As expected, PTSD was significantly more frequent in the high exposure group as compared to the low and the medium high groups.

The main finding of this study was a negative relationship between exposure to interpersonal violence in childhood and the FT3/FT4 ratio. Women with BPD exposed to interpersonal violence as a child had lower FT3/FT4 ratio. There may be a threshold of trauma exposure since the FT3/FT4 ratio was significantly lower in the high exposure group, with a KIVS scale score 4 or 5, indicating that only severe exposure to interpersonal violence as a child may be associated with an altered balance of peripheral deiodination. Interestingly, the comorbid PTSD diagnosis may have modified this relationship, the finding being significant only in patients with PTSD. Furthermore, in patients with PTSD, the exposure to interpersonal violence showed a significant negative correlation with the FT3 levels. To the best of our knowledge this is the first study measuring thyroid hormones in relation to reported exposure to interpersonal violence as a child in a large group of women with BPD.

Earlier studies with different clinical populations have reported a positive association between FT3/FT4 ratios and a history of childhood trauma. In a study by Girdler et al. (2004), women with premenstrual dysphoric disorder (PMDD) and a history of sexual abuse showed a greater FT3/FT4 ratio, as compared to women with PMDD and no history of sexual abuse as well as healthy controls. Friedman, Wang, Jalowiec, McHugo, and McDonagh-Coyle (2005) found significant elevations in TT3/FT4 and a significant reduction in TSH, in a community sample of 63 women with PTSD due to childhood sexual abuse as compared to 42 women without PTSD, of whom 17% also reported childhood sexual abuse.

As summarized in a report by Wang (2006), elevated T3 levels have earlier been reported in Vietnam, Israeli, World War II and Croatian combat veteran samples with PTSD. Studies of Vietnam veterans have reported elevated FT3/FT4 and TT3/FT4 ratios, in participants with PTSD, as compared to the non-PTSD group (Mason et al., 1994; Wang & Mason, 1999). In our study, levels of FT3 and FT3/FT4 ratio were higher, though not statistically significant in the comorbid PTSD group, as compared to the non-PTSD group.

There may be several reasons for the differences between the studies. First and foremost, the study populations differed with respect to gender, age and diagnosis. Secondly, the type, timing and duration of trauma exposure differed between the studies. The experience of stress elicits certain neurobiological mechanisms of in the HPT axis, with an increase of free thyroid hormones in the serum in conjunction with an increase of excretion via urine, leaving serum thyroid levels unchanged (Habermann et al., 1978) or within reference interval. KIVS measures different types of interpersonal violence including sexual violence. The nature of the trauma (physical or neglect), traumatic duration, setting of trauma, timing (i.e., stress exposure during certain developmental stages of the brain), and multiplicity of childhood traumata may evoke different mechanisms of stress regulation. Since the KIVS focus on interpersonal violence, neglect was not assessed in this present study. Longitudinal studies with stratification of traumatic exposure are sparse.

Severe and prolonged traumatic stress may expose the body and brain to physiological burdens in the stress-response systems at the level of both neurotransmitters as well as neuroendocrine interactions, leaving either permanent changes or shifting the body metabolism from homeostasis, into allostasis; a new adaptive physiological or behavioral state, maybe through epigenetic mechanisms. The impact of severe trauma may be likened to an allostatic overload, forcing the individual to adapt to new hormonal set points, in order to survive the demanding environment. This may be reflected in altered hormonal levels *within* normal reference ranges. Moreover, processes not contingent of thyroid hormone levels may have effect on regulation on thyroid hormone levels among individuals (Costa-e-Sousa & Hollenberg, 2012). These processes may include genetically determined tissue specific local deiodination and cell membrane thyroid hormone transport, genetic variation in Type 1 iodothyronine deiodinase (Dayan & Panicker, 2009), as well as neuronal circuitries of energy regulatory pathways in the HPT axis of higher order than the negative feedback system.

Interestingly, in the current study the cortisol levels were higher in women with PTSD. This may be in line with earlier studies indicating a dysregulation of HPA-axis in persons with PTSD (Meewisse et al., 2007). However, the high cortisol levels may also be the result of severity of BPD, comorbid depression, or the interaction between PTSD and BPD. In addition, individuals with BPD have been suggested to express hypercortisolemia in analogy with major depressive states (Zimmerman & Choi-Kain, 2009). We observed a weak but statistically significant relationship between sample storage time and FT3; therefore, we adjusted for sample storage time in the final regression model. However, the timing of blood sampling did not have a significant effect on hormone levels.

Among the limitations of this study is the cross-sectional study design, which prevents us from drawing casual conclusions. As the great majority of the patients were suffering from depression and had a history of severe suicidal behavior, we could not assess if the hormone levels were associated specifically to the depressive state or to the suicidal behavior. However, there were no significant differences in frequencies of antidepressants in different exposure groups. It is also worth mentioning that the study sample may not be representative of all BPD patients due to the specific selection criteria (i.e., history of at least two suicide attempts). Since the study was originally designed as a psychotherapy treatment study, it may be regarded as a convenience sample. Unfortunately, we did not have data of whether the women were in either luteal of follicular phase, which can be regarded as a limitation. Furthermore, three patients were taking lithium that is known to affect the thyroid hormone levels.

The strength of this study is the relatively large sample of women with thoroughly assessed DSM diagnosis of BPD. Furthermore, the study population is relatively homogenous with regard to age, gender, diagnosis and history of suicidal behavior, which should have lowered the variance due to unique adaptive metabolic mechanisms between individuals.

In summary, we found a negative relationship between exposure to interpersonal violence in childhood and the FT3/FT4, among 92 women with BPD. Comorbid diagnosis of PTSD was related to a more pronounced neuroendocrine dysregulation. It would be of great value to observe thyroid hormone changes in future longitudinal studies comprising individuals with and without PTSD, in order to capture time-related neuroendocrine variability.
